# Optimizing Dengue Surveillance Thresholds in Malaysia: A Comparative Evaluation of Endemic Channel Approaches

**DOI:** 10.3390/tropicalmed11080231

**Published:** 2026-08-19

**Authors:** Sarbhan Singh, Nuur Hafizah Md. Iderus, Lonny Chen Rong Qi Ahmad, Sumarni Mohd Ghazali, Nur’ain Mohd Ghazali, Mohd Nadzmi Md Nadzri, Asrul Anuar, Mohd Kamarulariffin Kamarudin, Lim Mei Cheng, Teh Chien Huey, Chong Zhuo Lin, Wan Ming Keong, Chew Cheng Hoon

**Affiliations:** 1 Institute for Medical Research (IMR), National Institutes of Health (NIH), Ministry of Health Malaysia, Setia Alam 40170, Malaysia; 2 Institute for Public Health (IPH), National Institutes of Health (NIH), Ministry of Health Malaysia, Setia Alam 40170, Malaysia; 3 Disease Control Division, Ministry of Health Malaysia, Putrajaya 62590, Malaysia

**Keywords:** dengue, endemic channel, surveillance, optimizing, multiplier, log scale

## Abstract

Endemic channels are widely used in dengue surveillance to detect unusual increases in case counts. However, conventional approaches that rely on historical averages with fixed standard deviation (SD)-based multipliers may produce unstable thresholds and false alerts. This study compared a conventional SD-based endemic channel with a log-scale SD-based endemic channel incorporating an enhanced alert rule to identify the optimal approach for routine dengue surveillance in Malaysia. Weekly national dengue case data from 2014 to 2024 were analyzed. A rolling validation approach was used to evaluate outbreak detection performance from 2017 to 2023 using three-year historical baselines. Sensitivity, specificity, positive predictive value, negative predictive value, accuracy, and the Youden Index were calculated and pooled across the validation years. The optimal multiplier was selected based on the highest pooled Youden Index using the rolling validation period (2017–2023). The selected approach was then applied independently to the 2024 surveillance data as an operational demonstration of its potential use in routine dengue surveillance. A total of 260 epidemiological weeks were analyzed, of which 42 (16.2%) were classified as outbreak weeks. The log-scale SD-based endemic channel incorporating an enhanced alert rule achieved the highest pooled Youden Index of 0.43 at the optimal multiplier of 0.50. At this multiplier, sensitivity was 0.60, specificity 0.82, positive predictive value 0.35, negative predictive value 0.93, and overall accuracy 0.79. Compared with the conventional SD-based endemic channel at its optimal multiplier (1.00), the proposed approach demonstrated improved sensitivity (0.60 vs. 0.56), specificity (0.82 vs. 0.70), positive predictive value (0.35 vs. 0.23), negative predictive value (0.93 vs. 0.91), accuracy (0.79 vs. 0.68), and Youden Index (0.43 vs. 0.26). The log-scale SD-based endemic channel incorporating an enhanced alert rule demonstrated superior overall outbreak detection performance and was selected as the optimal approach for routine dengue surveillance.

## 1. Introduction

Dengue fever, caused by the dengue virus (DENV) and transmitted primarily by *Aedes aegypti* and *Aedes albopictus*, remains one of the most important vector-borne diseases globally. Endemic in more than 100 countries, dengue places nearly half of the world’s population at risk and contributes substantially to morbidity, mortality, and healthcare burden, particularly in tropical and subtropical regions [1]. The continuing rise in dengue incidence worldwide highlights the need for surveillance systems capable of supporting timely outbreak detection and effective public health response.

In Malaysia, dengue has remained endemic for decades, with marked year-to-year variation in incidence. National case counts exceeded 100,000 in 2014, peaked at more than 130,000 cases in 2019, declined during the COVID-19 pandemic period, and resurged thereafter, with 2024 recording one of the highest annual dengue cases [2]. The COVID-19 pandemic substantially altered dengue transmission dynamics in Malaysia through multiple mechanisms, including movement restrictions, changes in human mobility, modifications in healthcare-seeking behavior, altered vector–human contact patterns, and potential changes in case reporting. Consequently, dengue incidence during 2020 and 2021 differed considerably from historical transmission patterns and did not represent the underlying endemic epidemiology observed before or after the pandemic. These fluctuations reflect the complex and dynamic nature of dengue transmission, influenced by seasonality, long-term trends, and external disruptions.

Surveillance thresholds represent predefined statistical boundaries that distinguish expected background disease activity from unusual increases that may indicate the early stages of an outbreak. Rather than serving solely as statistical cut-offs, these thresholds function as operational decision-support tools by identifying when disease activity exceeds what would normally be expected based on historical patterns. In routine dengue surveillance, threshold exceedance may trigger a range of public health actions, including verification of reported increases, epidemiological investigation, intensified vector surveillance, source reduction activities, larviciding and fogging where appropriate, enhanced risk communication, increased clinical vigilance, and mobilization of outbreak response resources. Consequently, the timely identification of abnormal increases in dengue transmission is essential for enabling early intervention, reducing onward transmission, and minimizing disease burden.

However, establishing reliable surveillance thresholds for dengue remains challenging. Dengue transmission exhibits substantial temporal variability and overdispersion, making it difficult to distinguish expected seasonal fluctuations from epidemiologically meaningful increases that warrant public health action. Although outbreaks are generally identified when case counts exceed expected levels, defining these thresholds in a reliable and operationally meaningful manner remains a significant challenge. Several approaches have been proposed for dengue surveillance, including incidence-based indicators, infectivity measures, and endemic channels, each with distinct methodological strengths and limitations [3,4,5].

More recently, data-driven and artificial intelligence (AI)-based models have been explored for dengue early warning by integrating climatic, environmental, entomological, and other surveillance data to improve outbreak prediction. However, these approaches often require large, high-quality datasets and greater computational complexity, which may limit their routine implementation. Consequently, endemic channels remain widely used because they are simple, transparent, and operationally feasible. Improving the performance of these conventional surveillance tools therefore remains an important public health priority.

Despite these operational advantages, conventional endemic channel estimation approaches have several important methodological limitations. These methods typically rely on historical weekly means and fixed standard deviation (SD)-based multipliers, with the choice of multiplier often based on convention rather than empirical optimization or validation against outbreak detection performance [6,7,8]. Consequently, their performance is not inherently too sensitive or too specific but varies considerably depending on the characteristics of the surveillance data and the selected threshold multiplier. Furthermore, because these approaches rely solely on threshold exceedance, they are susceptible to short-term random fluctuations that may generate false alerts. Their performance may also be distorted by atypical epidemic years; unusually large outbreaks can inflate historical means and standard deviations, resulting in thresholds that become less responsive in subsequent years, whereas periods of unusually low transmission may produce thresholds that are overly sensitive and increase false-positive alerts. Although endemic channels remain attractive because of their simplicity, transparency, and ease of implementation, their operational reliability and outbreak detection performance remain uncertain, particularly in endemic settings with evolving transmission dynamics such as Malaysia.

Therefore, this study focused on a practical modification of the conventional SD-based endemic channel that incorporates log-scale transformation and an enhanced alert rule. Log-scale transformation is commonly applied in epidemiological time-series data to stabilize variance and reduce the influence of extreme values. The enhanced alert rule, which requires sustained increases in weekly case counts rather than isolated threshold exceedance alone, may help reduce false alerts [8,9,10]. This approach remains relatively simple to implement while addressing some limitations of the conventional SD-based endemic channel. However, it has not been systematically evaluated against the conventional endemic channel approach in the local dengue surveillance context.

In this study, we compare a conventional SD-based endemic channel approach with a log-scale SD-based endemic channel incorporating an enhanced alert rule. Performance is assessed across multiple time periods to evaluate the consistency of outbreak detection and to identify the optimal endemic channel approach. The selected approach was subsequently applied to 2024 dengue surveillance data as an operational demonstration of its potential use in routine public health practice.

## 2. Materials and Methods

### 2.1. Data Source

Dengue case data from 2014 to 2024 were obtained from the national dengue surveillance database of the Disease Control Division, Ministry of Health, Malaysia. Under the Prevention and Control of Infectious Diseases Act 1988 (Act 342), all clinically suspected and laboratory-confirmed dengue cases are mandatorily reported within 24 h to the nearest health authority [11]. A confirmed dengue case meets both clinical and laboratory criteria as defined by national guidelines [11]. Owing to the substantial disruption in dengue transmission patterns during the COVID-19 pandemic, data from 2020 and 2021 were excluded to ensure that the historical baseline reflected typical endemic transmission dynamics. Analyses were conducted using national weekly dengue surveillance data representing the whole of Malaysia.

### 2.2. Reference Outbreak Definition

Weekly outbreak status was defined using an operational proxy indicator reflecting sustained increases in dengue transmission. The reference outbreak definition was developed based on published dengue early warning literature and refined following consultation with subject matter experts, recognizing that no universally accepted gold standard exists for defining dengue outbreak onset. An outbreak week was defined when both of the following conditions were met: (a) two consecutive increases in weekly dengue case counts and (b) a minimum 10% increase compared with the value two weeks earlier [12,13]. The requirement for consecutive increases was intended to reduce misclassification arising from short-term random fluctuations, while the relative increase criterion was included to ensure that detected signals reflected meaningful increases in transmission rather than minor stochastic variation. The 10% threshold was selected as a pragmatic operational cut-off balancing sensitivity to emerging outbreaks with robustness against random variation. Accordingly, the reported performance measures should be interpreted as relative comparisons between surveillance approaches under a common operational reference framework rather than absolute measures of outbreak detection accuracy.

### 2.3. Endemic Channel Estimation Approach

Two endemic channel approaches were evaluated as follows:

Approach 1. Conventional SD-Based Endemic Channel Estimation

Weekly endemic channels were constructed for each year from 2017 to 2023 using a three-year rolling historical baseline [4,14,15]. For each epidemiological week, dengue case counts from the preceding three years were used to calculate the week-specific mean and SD. The endemic channel was defined as follows:

*EC* = *μ* + *mσ*

where

• EC = endemic channel;
• μ = mean weekly dengue case count from the historical baseline;
• σ = standard deviation of weekly dengue case counts from the historical baseline;
• m = multiplier.

An outbreak alert was generated when the observed weekly dengue case count exceeded the endemic threshold for that epidemiological week. No additional growth or persistence condition was applied. The mean plus standard deviation approach has been widely adopted because it provides a simple and transparent method for estimating expected disease activity using routinely collected surveillance data. The approach is computationally straightforward, requires minimal statistical expertise, and can be readily implemented within routine public health surveillance systems without specialized software or complex modeling assumptions. Consequently, it has been widely adopted by national dengue programs and recommended within WHO operational guidance for endemic channel construction.

Approach 2. Log-scale SD-Based Endemic Channel with Enhanced Alert Rule

Weekly endemic channels were constructed for each validation year from 2017 to 2023 using the same three-year rolling historical baseline. Weekly dengue case counts from the historical baseline were transformed using the natural logarithm after adding one to each observation [ln(x + 1)] to stabilize variance, reduce the influence of highly skewed epidemic observations, and retain weeks with zero reported cases [16]. For each epidemiological week, the mean and standard deviation (SD) of the transformed values were calculated across the historical baseline.

The log-transformed endemic channel was estimated as follows:

*EC*_log_ = *μ*_ln (_*_x_*_ + 1)_ + *mσ_l_*_n (_*_x_*_ + 1)_

where

• EC_log_ = endemic channel on the log-transformed scale;
• x=weekly case counts;
• μ_ln (__x__ + 1)_ = mean of the natural log-transformed weekly dengue case counts;
• σ_ln (__x__ + 1)_ = standard deviation of the natural log-transformed weekly dengue case counts;
• m=multiplier.

The estimated threshold was subsequently back-transformed to the original case-count scale using the exponential function:

*EC* = exp (EC_log_) − 1

Because the threshold was estimated on the logarithmic scale, the back-transformed threshold represents a geometric estimate rather than an arithmetic mean-based threshold and is therefore less influenced by extreme epidemic observations.

To evaluate the independent contribution of logarithmic transformation, the log-scale SD-based endemic channel was first evaluated without the enhanced alert rule. This additional analysis was performed solely to assess the incremental effect of logarithmic transformation before incorporation of the enhanced alert rule. Subsequently, an enhanced alert rule was applied, whereby an outbreak alert was generated only when both of the following conditions were satisfied: (a) the observed weekly dengue case count exceeded the back-transformed endemic threshold, and (b) the observed weekly dengue case count was higher than that of the preceding epidemiological week [17].

The enhanced alert rule was defined as follows:

Alert if *Y_t_* > *EC_t_* and *Y_t_* > *Y_t_*_−__1_

where

• Y_t_ = observed weekly dengue case count at epidemiological week t;
• EC_t_ = back-transformed endemic channel threshold at epidemiological week t.

The enhanced alert rule was incorporated to reduce false-positive alerts arising from isolated threshold exceedance by requiring evidence of sustained upward transmission before generating an outbreak signal.

### 2.4. Performance Evaluation and Selection of the Optimal Approach

The rolling validation period (2017–2023) was used to evaluate model performance and identify the optimal multiplier [18,19]. A three-year rolling historical baseline was used for endemic channel construction throughout the validation period. For validation years from 2017 to 2019, the baseline comprised data from the three immediately preceding years. Because dengue transmission patterns during 2020 and 2021 were substantially disrupted by the COVID-19 pandemic and associated public health interventions, these years were excluded from the historical baseline to avoid distortion of the endemic thresholds. Consequently, the historical baseline for the 2022 validation year comprised data from 2017 to 2019, whereas the baseline for 2023 comprised data from 2018, 2019, and 2022. The historical baseline used for each validation year is summarized in Table 1.

For each test year, predicted outbreak alerts generated using the corresponding historical baseline were compared with the reference outbreak definition. Sensitivity (SN), Specificity (SP), Positive Predictive Value (PPV), Negative Predictive Value (NPV), Accuracy, and the Youden Index were calculated for each test year.

In the context of dengue surveillance, SN reflects the ability to identify outbreak weeks, whereas SP reflects the ability to avoid unnecessary outbreak alerts during periods of normal transmission. PPV indicates the probability that an alert corresponds to an outbreak week, while NPV reflects the probability that the absence of an alert corresponds to a non-outbreak week. Accuracy summarizes the overall proportion of correctly classified weeks but may be influenced by the relatively low prevalence of outbreak weeks. The Youden Index provides a prevalence-independent summary measure of discrimination by combining sensitivity and SP and was therefore used as the primary optimization criterion. Because no single performance metric adequately reflects overall surveillance performance, multiple complementary measures were evaluated. Overall performance was described using pooled SN, SP, PPV, NPV, accuracy, and Youden Index. The objective of the optimization process was to identify the threshold multiplier that provided the best overall discrimination between outbreak and non-outbreak weeks. Accordingly, the multiplier with the highest pooled Youden Index was considered optimal, representing the best overall balance between early outbreak detection and minimizing false-positive alerts across all validation years.

To assess the precision and consistency of the performance estimates, pooled performance measures were calculated by aggregating the true positives, true negatives, false positives, and false negatives across all validation years for each multiplier to form pooled confusion matrices. SN, SP, PPV, NPV, accuracy, and the Youden Index were subsequently calculated from these pooled confusion matrices rather than by averaging yearly performance estimates. Ninety-five percent confidence intervals (95% CIs) for SN, SP, PPV, NPV, and accuracy were calculated using the Wilson score method, whereas the 95% CI for the Youden Index was estimated using non-parametric bootstrap resampling with 2000 iterations. In addition, year-specific performance metrics were summarized for each validation year to assess the consistency of performance across the rolling validation periods. These results are presented in the Supplementary Materials (Tables S1 and S2).

### 2.5. Application of the Selected Approach to Routine Surveillance

The selected endemic channel approach and its optimal multiplier, identified during the 2017–2023 validation period, were subsequently applied to the 2024 dengue surveillance data. The 2024 data were not used for model development, validation, or selection but were analyzed independently as an operational demonstration of the selected approach in a routine surveillance setting. The resulting endemic thresholds were plotted against the observed weekly dengue case counts to illustrate the practical application of the optimized approach for outbreak monitoring.

### 2.6. Software

All analyses were conducted using R version 4.6.1. Data processing and modeling were performed using packages including readxl, dplyr, caret, ggplot2, binom, and writexl.

## 3. Results

A total of 260 epidemiological weeks were evaluated, of which 42 (16.2%) were classified as outbreak weeks based on the reference outbreak definition.

### 3.1. Endemic Channel Estimation Approach

Approach 1. Conventional SD-Based Endemic Channel

The performance of the conventional SD-based endemic channel across evaluated multipliers is presented in Table 2. SN was highest at lower multipliers, remaining at 0.60 for multipliers between 0.00 and 0.50, and decreased progressively as the multiplier increased, reaching 0.26 at a multiplier of 2.00. In contrast, SP increased from 0.50 at a multiplier of 0.00 to 0.89 at a multiplier of 2.00. PPV increased modestly from 0.16 to 0.27, while NPV remained consistently high, ranging from 0.88 to 0.91. Overall accuracy increased with higher multipliers, from 0.51 to 0.80. The highest Youden Index was 0.26, observed at a multiplier of 1.00.

Approach 2. Log-scale SD-Based Endemic Channel with Enhanced Alert Rule

The performance of the log-scale SD-based endemic channel with the enhanced alert rule across evaluated multipliers is presented in Table 3. SN ranged from 0.60 at lower multipliers (0.00 to 0.50) to 0.23 at a multiplier of 2.00, with a gradual decrease observed as the multiplier increased. SP increased with increasing multipliers, ranging from 0.76 at a multiplier of 0.00 to 0.98 at a multiplier of 2.00. PPV increased from 0.29 at a multiplier of 0.00 to 0.67 at a multiplier of 2.00. NPV remained consistently high across multipliers, ranging from 0.89 to 0.93. Overall accuracy increased from 0.74 at a multiplier of 0.00 to 0.88 at a multiplier of 2.00. The highest Youden Index was 0.43, observed at a multiplier of 0.5.

Compared with the conventional SD-based endemic channel, logarithmic transformation alone improved SN (0.56 vs. 0.58), SP (0.70 vs. 0.75), PPV (0.23 vs. 0.28), accuracy (0.68 vs. 0.72), and the Youden Index (0.26 vs. 0.33). Incorporation of the enhanced alert rule further improved surveillance performance, resulting in the highest overall Youden Index of 0.43.

### 3.2. Performance Evaluation and Selection of the Optimal Approach

**Figure 1:**
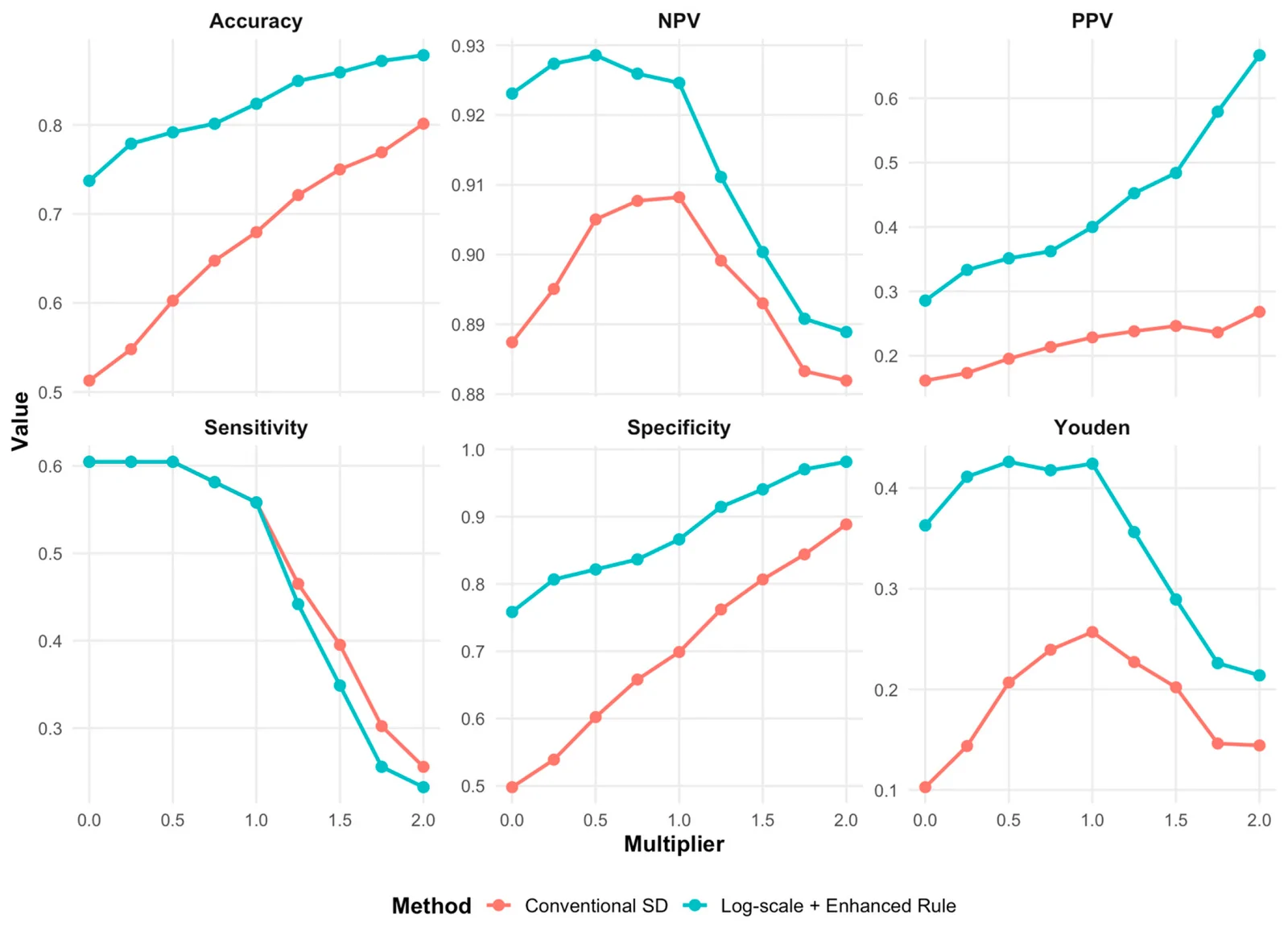
Comparison of endemic channel performance across evaluated multipliers for the conventional SD-based endemic channel and the log-scale SD-based endemic channel with an enhanced alert rule.

Across evaluated multipliers, the log-scale SD-based endemic channel with the enhanced alert rule demonstrated higher SP, PPV, overall accuracy, and Youden Index than the conventional SD-based endemic channel (Figure 1). The best-performing conventional SD-based approach achieved a pooled Youden Index of 0.26 at a multiplier of 1.00, while the best-performing log-scale enhanced approach achieved a higher pooled Youden Index of 0.43 at a multiplier of 0.50. Therefore, the log-scale SD-based endemic channel with the enhanced alert rule was selected as the optimal approach.

To clarify the independent contribution of logarithmic transformation and the enhanced alert rule, an additional comparative analysis was performed using the conventional SD-based endemic channel, the log-scale SD-based endemic channel, and the log-scale SD-based endemic channel with the enhanced alert rule (Table 4). Logarithmic transformation alone produced modest performance improvements compared with the conventional SD-based endemic channel. At the optimal multiplier of 1.00, SN increased from 0.56 to 0.58, SP from 0.70 to 0.75, PPV from 0.23 to 0.25, NPV from 0.91 to 0.92, accuracy from 0.68 to 0.70, and the Youden Index from 0.26 to 0.28. Following the incorporation of the enhanced alert rule, endemic channel performance improved substantially across all evaluated performance measures. At an optimal multiplier of 0.50, SN increased to 0.60, SP to 0.82, PPV to 0.35, NPV to 0.93, accuracy to 0.79, and the Youden Index to 0.43. These findings demonstrate that both logarithmic transformation and the enhanced alert rule independently contributed to the improved endemic channel performance, with the enhanced alert rule providing the greater incremental improvement.

**Figure 2:**
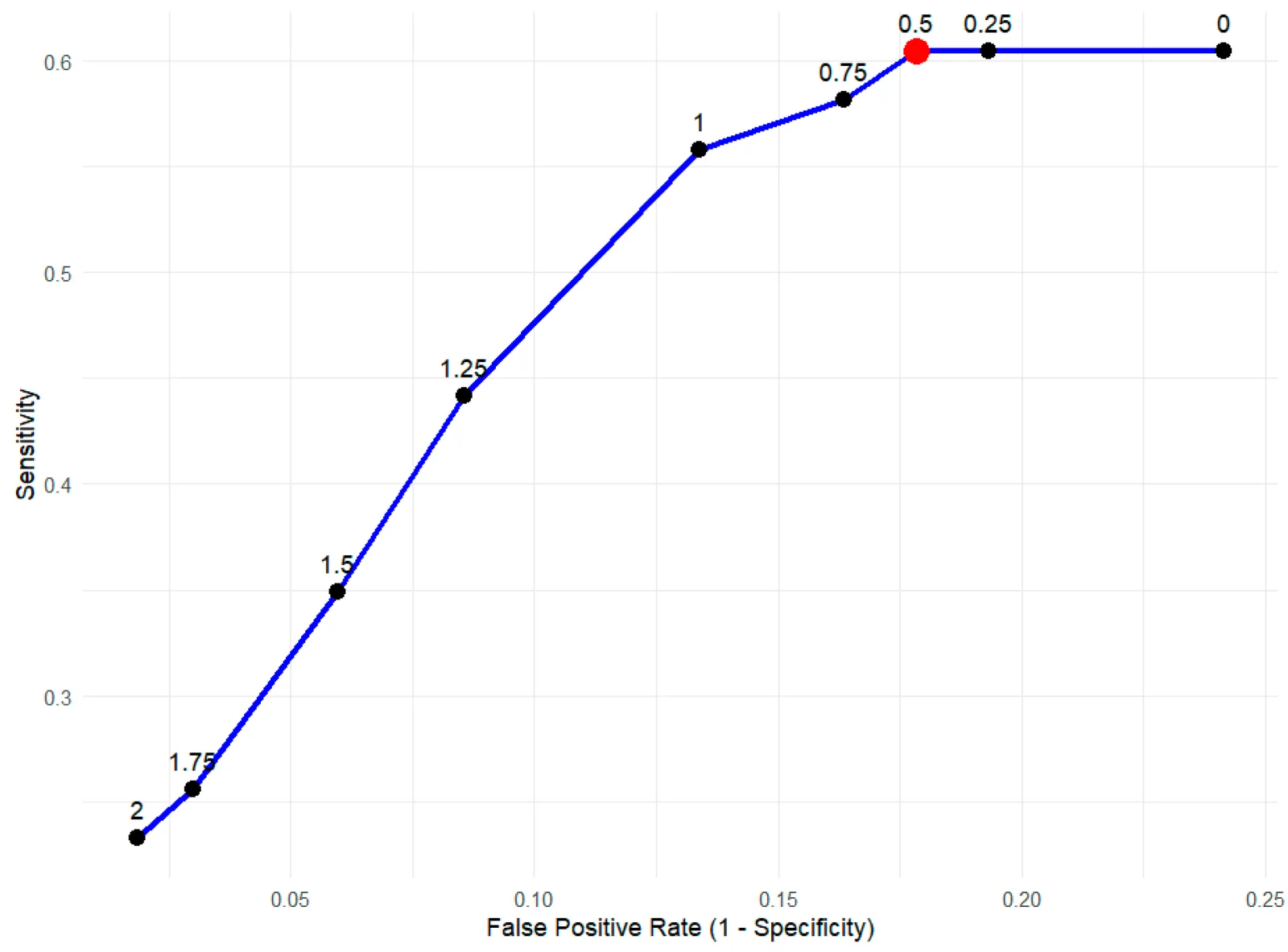
SN versus false-positive rate across multipliers for the log-scale endemic channel (optimal multiplier = 0.50).

For the selected log-scale enhanced approach, the optimal pooled performance was observed at a multiplier of 0.50. At this multiplier, SN was 0.60, SP was 0.82, PPV was 0.35, NPV was 0.93, and overall accuracy was 0.79. The trade-off between SN and false-positive rate across candidate multipliers is shown in Figure 2.

### 3.3. Application of the Selected Approach to 2024 Dengue Surveillance

**Figure 3:**
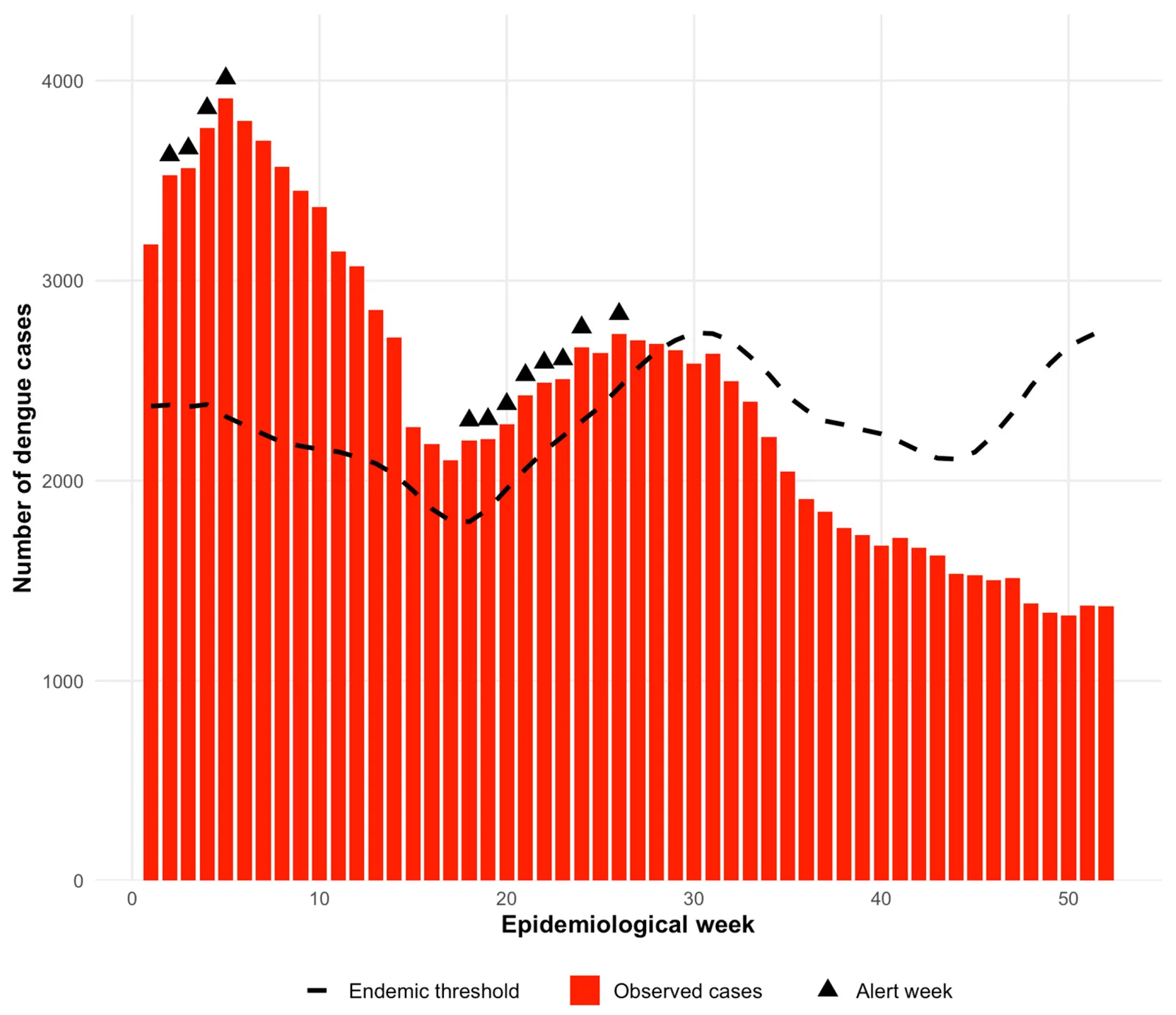
Observed weekly dengue cases and endemic threshold, Malaysia, 2024.

The selected log-scale SD-based endemic channel with the enhanced alert rule was applied to 2024 dengue case data using the optimal multiplier identified from pooled validation analysis (multiplier = 0.50). The resulting endemic threshold was plotted alongside observed weekly dengue case counts across epidemiological weeks (Figure 3). The observed weekly dengue case counts exceeded the endemic threshold during the early epidemiological weeks of 2024, particularly from approximately Week 2 to Week 15. Case counts then declined and remained closer to, or below, the endemic threshold during the middle of the year. From approximately Week 34 onwards, observed case counts remained below the endemic threshold until the end of the year. The endemic threshold varied across epidemiological weeks, reflecting the week-specific baseline estimated from historical dengue case patterns.

## 4. Discussion

This study evaluated the performance of a conventional SD-based endemic channel against a refined log-scale SD-based endemic channel incorporating an enhanced alert rule for optimizing dengue surveillance thresholds in Malaysia. Using a rolling validation framework, the findings demonstrate that the log-scale approach consistently achieved higher SP, PPV, overall accuracy, and Youden Index across evaluated multipliers while maintaining comparable SN at lower threshold levels.

The improved performance of the log-scale approach is likely attributable to its ability to better accommodate the statistical properties of dengue case data. Dengue cases are typically characterized by overdispersion and right-skewed distributions, particularly during epidemic peaks. Log transformation has been widely applied in epidemiological analyses to stabilize variance and reduce the influence of extreme values, thereby improving the robustness of statistical estimation [16]. By reducing the influence of large outbreak spikes on baseline estimation, the log-scale approach provides a more stable reference for estimation of dengue-endemic channels compared with the conventional approach. The additional component analysis demonstrated that logarithmic transformation alone improved surveillance performance compared with the conventional SD-based endemic channel, while subsequent incorporation of the enhanced alert rule resulted in further improvements across all evaluated performance measures. These findings suggest that both variance stabilization through logarithmic transformation and the enhanced alert rule independently contributed to the observed improvement in outbreak detection performance.

The enhanced alert rule, which requires both threshold exceedance and week-to-week increase in case counts, appears to improve discrimination between sustained transmission and short-term random fluctuations. The conventional endemic channel approach typically relies on threshold exceedance alone, which may generate false alerts in the presence of random variation. The inclusion of a temporal condition aligns with outbreak detection definitions that emphasize persistence and trend in identifying meaningful signals [17]. Improved outbreak detection has the potential to support earlier epidemiological investigations and more timely implementation of routine vector control activities, including source reduction, larviciding, targeted space spraying (fogging), environmental sanitation, and community engagement. However, the present study evaluated surveillance performance rather than operational vector control outcomes.

A key contribution of this study is the application of a rolling validation framework to evaluate endemic channel performance across multiple time periods. Unlike traditional approaches that apply fixed thresholds without formal validation, the rolling origin design preserves temporal independence by ensuring that only historical data are used to generate predictions for each test year. This approach more closely reflects real-world surveillance practices and minimizes optimistic bias associated with retrospective model evaluation. The observed variability in optimal multipliers across test years highlights the dynamic nature of dengue transmission and the limitations of relying on fixed thresholds. To identify an operationally robust threshold suitable for routine surveillance, performance was aggregated across all validation years, and the optimal multiplier was selected based on the highest pooled Youden Index, representing the best overall balance between SN and SP. To our knowledge, this is among the first studies to systematically integrate variance stabilization, temporal alert criteria, and rolling validation within an endemic channel framework and evaluate their combined performance.

The findings of this study are consistent with existing literature highlighting the challenges of defining outbreak thresholds in dengue surveillance. Previous work has demonstrated that outbreak definitions are inherently variable and context-dependent, with no universally accepted gold standard [13]. Similarly, multi-country studies of dengue early warning systems, including modern forecasting approaches, have relied on proxy indicators and alarm variables rather than definitive outbreak labels [19]. In this context, the use of a rule-based proxy definition in the present study is consistent with established practice in early warning system development. Rather than representing a definitive outbreak classification, the reference outbreak definition served as a pragmatic operational benchmark for comparing the relative performance of alternative surveillance approaches. Importantly, the same reference outbreak definition was applied consistently across all evaluated approaches, allowing for a fair comparison of relative performance.

In Malaysia, routine dengue case surveillance forms a fundamental component of the national dengue prevention and control program. Surveillance data are routinely used to trigger epidemiological investigations and guide vector control activities, including environmental management, larval control, adult mosquito control, and community engagement. Consequently, the timeliness and reliability of surveillance alerts are important for prioritizing limited public health resources and supporting timely implementation of vector control interventions. By reducing false-positive alerts while maintaining comparable SN, the proposed endemic channel approach has the potential to improve operational decision-making by directing vector control resources towards periods of increased transmission risk. Although the present study did not directly evaluate operational vector control outcomes, improved surveillance performance may support more targeted deployment of routine dengue control measures.

Building upon this operational context, the prospective application of the optimized endemic channel to the 2024 surveillance data illustrates how the selected approach could be incorporated into routine dengue surveillance. Because independent outbreak declarations or operational public health records were not available for comparison, the 2024 application should be interpreted as an operational demonstration rather than a formal prospective validation. The visualization of observed case counts alongside the endemic threshold provides an interpretable tool for identifying periods of increased transmission. Such surveillance thresholds may support timely outbreak verification, guide epidemiological investigations, prioritize vector control activities, facilitate more efficient allocation of public health resources, and enhance situational awareness during periods of increased dengue transmission. Although this study did not evaluate the operational impact of threshold-guided interventions, the proposed approach has the potential to strengthen routine surveillance by supporting earlier and more informed public health decision-making.

Although the PPV of the selected approach remained modest (0.35), surveillance thresholds should be interpreted as early warning tools rather than definitive outbreak declaration tools. In the present study, only 42 of 260 epidemiological weeks (16.2%) were classified as outbreak weeks according to the reference outbreak definition, resulting in a relatively low prevalence of outbreak weeks that inherently influenced the PPV. Under these circumstances, a proportion of false-positive alerts is expected and may represent an acceptable trade-off for improving the timely detection of potential outbreaks and facilitating early public health investigation and intervention. Importantly, despite the modest PPV, the proposed approach demonstrated higher PPV than the conventional endemic channel while maintaining improved overall discrimination. Consequently, alerts generated by the proposed endemic channel should be regarded as signals warranting further epidemiological assessment and operational review, rather than definitive confirmation of an outbreak. Such alerts should be interpreted alongside other surveillance information, including epidemiological investigations, laboratory findings, entomological indicators, and local situational context before an outbreak is formally declared.

Several limitations should be considered. First, the reference outbreak definition was based on a proxy indicator derived from surveillance data rather than an independently verified gold standard. As a result, the reported performance metrics reflect agreement with the proxy definition rather than absolute outbreak detection accuracy. However, this approach is consistent with the inherent challenges in defining dengue outbreak onset and aligns with previous studies in dengue surveillance [12,13]. Importantly, the reference outbreak definition was developed based on published dengue surveillance literature and refined following consultation with subject matter experts. Nevertheless, because the enhanced alert rule also incorporates a temporal increase criterion, this partial overlap may have favored the proposed approach. Consequently, the reported performance measures should be interpreted as relative comparisons under a common operational reference framework rather than absolute measures of outbreak detection accuracy. Second, the analysis was conducted using nationally aggregated data, which may mask spatial heterogeneity in dengue transmission patterns. Subnational analyses may yield different optimal thresholds and should be explored in future studies. Third, the relatively small proportion of outbreak weeks may influence the interpretation of accuracy, PPV, and NPV. Therefore, surveillance performance was evaluated using multiple complementary performance measures, and approach selection was based primarily on the Youden Index rather than accuracy alone. Finally, excluding the COVID-19 years from the rolling validation resulted in a non-contiguous historical baseline. However, this was intended to minimize bias arising from atypical dengue transmission patterns during the pandemic. Inclusion of these years would likely have resulted in artificially depressed baseline thresholds, thereby increasing the probability of false outbreak alerts during the post-pandemic period when dengue incidence returned towards pre-pandemic levels.

Despite these limitations, this study provides a structured and empirically grounded approach for evaluating endemic channel performance. By integrating variance stabilization, temporal alert criteria, and rolling validation, the proposed approach addresses key limitations of conventional endemic channels while remaining compatible with routine surveillance systems. Although the proposed approach improved the performance of conventional endemic channels, fixed-threshold surveillance methods remain less adaptive than modern data-driven and AI-based forecasting approaches that incorporate dynamic epidemiological, climatic, environmental, and entomological information [20]. Nevertheless, endemic channels remain valuable because of their simplicity, transparency, and operational feasibility and should be viewed as complementary components of dengue surveillance rather than replacements for more advanced forecasting systems. Future studies should also evaluate the proposed approach against independent operational outbreak reference standards, such as official outbreak declarations, laboratory-confirmed outbreak investigations, or documented implementation of outbreak control measures. Future research should further evaluate the proposed approach at subnational levels across different geographical regions and climatic seasons, where dengue transmission dynamics may vary considerably. In addition, integrating complementary data sources, such as climatic, entomological, and vector surveillance indicators, may further improve seasonal calibration of surveillance thresholds and strengthen outbreak detection capability.

Taken together, the findings of the present study demonstrate that the proposed log-scale SD-based endemic channel incorporating the enhanced alert rule demonstrated meaningful improvements over the conventional SD-based endemic channel. Compared with the conventional approach, the proposed method improved SN (0.60 vs. 0.56), SP (0.82 vs. 0.70), PPV (0.35 vs. 0.23), overall accuracy (0.79 vs. 0.68), and the Youden Index (0.43 vs. 0.26). These improvements indicate a more favorable balance between early outbreak detection and reduction in false-positive alerts. In the context of routine dengue surveillance, more reliable outbreak alerts may support earlier epidemiological investigation and more efficient prioritization of vector control activities, including source reduction, larviciding, targeted space spraying (fogging), environmental sanitation, and community engagement. Although the present study did not evaluate operational vector control outcomes, the proposed approach represents a simple, transparent, and operationally feasible enhancement to conventional endemic channel surveillance and provides a practical framework for strengthening routine dengue surveillance in endemic settings.

## 5. Conclusions

This study demonstrates that the log-scale SD-based endemic channel incorporating an enhanced alert rule and evaluated using a rolling validation framework provides improved performance compared to the conventional approach for endemic channel estimations. The refined approach achieved a more favorable balance between SN and SP, with reduced false-alert rates and improved overall discrimination. The identification of an empirically optimized multiplier and its prospective application to national dengue data highlight the practical utility of the methods for routine surveillance. By addressing key methodological limitations in the conventional endemic channel approach, this approach offers a data-driven and operationally feasible tool for early warning systems in dengue-endemic settings.

## Figures and Tables

**Table 1:** Test year and corresponding historical baseline used.

Test Year	Historical Baseline Used
2017	2014–2016
2018	2015–2017
2019	2016–2018
2022	2017–2019
2023	2018, 2019, 2022

**Table 2:** Performance of the conventional SD-based endemic channel across evaluated multipliers.

Multiplier	SN	SP	PPV	NPV	Accuracy	Youden
0.00	0.60	0.50	0.16	0.89	0.51	0.10
0.25	0.60	0.54	0.17	0.90	0.55	0.14
0.50	0.60	0.60	0.20	0.91	0.60	0.21
0.75	0.58	0.66	0.21	0.91	0.65	0.24
1.00	0.56	0.70	0.23	0.91	0.68	0.26
1.25	0.47	0.76	0.24	0.90	0.72	0.23
1.50	0.40	0.81	0.25	0.89	0.75	0.20
1.75	0.30	0.84	0.24	0.88	0.77	0.15
2.00	0.26	0.89	0.27	0.88	0.80	0.14

Note: 95% confidence intervals for all performance estimates are provided in Supplementary Table S2.

**Table 3:** Performance of log-scale SD-based endemic channel with enhanced alert rule across evaluated multipliers.

Multiplier	SN	SP	PPV	NPV	Accuracy	Youden
0.00	0.60	0.76	0.29	0.92	0.74	0.36
0.25	0.60	0.81	0.33	0.93	0.78	0.41
0.50	0.60	0.82	0.35	0.93	0.79	0.43
0.75	0.58	0.84	0.36	0.93	0.80	0.42
1.00	0.56	0.87	0.40	0.92	0.82	0.42
1.25	0.44	0.91	0.45	0.91	0.85	0.36
1.50	0.35	0.94	0.48	0.90	0.86	0.29
1.75	0.26	0.97	0.58	0.89	0.87	0.23
2.00	0.23	0.98	0.67	0.89	0.88	0.21

Note: 95% confidence intervals for all performance estimates are provided in Supplementary Table S2.

**Table 4:** Comparison of endemic channel performance among the conventional SD-based endemic channel, the log-scale SD-based endemic channel, and the log-scale SD-based endemic channel with the enhanced alert rule at their optimal multipliers.

Method	Multiplier	SN	SP	PPV	NPV	Accuracy	Youden
Conventional SD	1.00	0.56	0.70	0.23	0.91	0.68	0.26
Log-scale SD	1.00	0.58	0.75	0.25	0.92	0.70	0.28
Log-scale SD with Enhanced Rule	0.50	0.60	0.82	0.35	0.93	0.79	0.43

## Data Availability

The datasets presented in this article are not publicly available, as they comprise national-level dengue surveillance data and require prior approval from the Ministry of Health Malaysia for disclosure. The analytic code underpinning the endemic channel estimation and validation framework is available from the authors upon reasonable request and should be directed to lssarbhan@moh.gov.my.

## Supplementary Materials

The following supporting information can be downloaded at https://www.mdpi.com/article/10.3390/tropicalmed11080231/s1, Supplementary Table S1. Year-specific diagnostic performance of the evaluated endemic channel approaches across the rolling validation period (2017–2023). Supplementary Table S2. Pooled diagnostic performance of the evaluated endemic channel approaches with 95% confidence intervals.

## Abbreviations

The following abbreviations are used in this manuscript:

SD	Standard Deviation
SN	Sensitivity
SP	Specificity
PPV	Positive Predictive Value
NPV	Negative Predictive Value
